# The role of the thalamus in focal human epilepsy: insights from stereoelectroencephalography (SEEG)

**DOI:** 10.3389/fneur.2025.1608715

**Published:** 2025-06-02

**Authors:** Odile Feys, Francesca Pizzo, Julia Makhalova, Romain Carron, Fabrice Bartolomei

**Affiliations:** ^1^APHM, Timone Hospital, Epileptology and Cerebral Rhythmology, Marseille, France; ^2^Aix-Marseille Université, INSERM, INS, Institut de Neurosciences des Systèmes, Marseille, France; ^3^Aix-Marseille University, CNRS, CRMBM, Marseille, France; ^4^Timone Hospital, Medico-Surgical Unit Epileptology, Functional and Stereotactic Neurosurgery, Marseille, France

**Keywords:** refractory epilepsy, thalamic epileptogenicity, stereotactic techniques, thalamic nuclei, subcortical areas

## Abstract

This review explores the role of the thalamus in focal epilepsy, focusing on insights gained from stereoelectroencephalography (SEEG). The thalamus has recently regained attention as a crucial player in seizure dynamics. Thalamic SEEG recordings can be used to assess certain aspects of the thalamus’s role in human focal epilepsy, in particular the timing and dynamics of involvement of distinct thalamic nuclei during seizures and in interictal activity. Estimation of thalamic involvement in seizure propagation may be valuable before embarking on surgical resection and provide guidance for neuromodulation strategies. High thalamic epileptogenicity correlates with poorer surgical outcomes, making it a predictive biomarker. Deep brain stimulation (DBS), particularly targeting the anterior and pulvinar nuclei, has effectively reduced seizure frequency and improved consciousness during seizures. However, the effectiveness of DBS varies, emphasizing the need for individual targeting based on individual seizure dynamics. High-frequency thalamic stimulation can reduce seizure frequency and alter epileptogenic networks, offering tailored therapeutic approaches. Despite the potential added surgical risks of depth electrode implantation, thalamic SEEG significantly enhances the understanding of epileptogenic networks. It supports the development of personalized epilepsy treatments by elucidating the complex interplay between cortical and subcortical regions, paving the way for improved seizure management and neuromodulation strategies.

## Introduction

The role of the thalamus in the genesis and propagation of spikes-waves discharges is well known in generalized epilepsies, particularly in the pathophysiology of absence seizures (1–3). This subcortical region has been historically rarely investigated by stereo-electroencephalography (SEEG), compared with cortical areas, due to the lack of established guidelines for its coverage (4). Nonetheless, it has been experimentally shown that thalamic connectivity impacts the propagation of temporal lobe seizures (5). Introduced in the sixties, the SEEG method was developed as a presurgical tool to investigate focal epilepsies and provides a unique means to investigate the role of subcortical regions, including the thalamus, in the genesis and dynamic of seizures (6). The first reports on the thalamic SEEG recordings during seizures stem from French groups and date back to 2006 (7, 8). Since then, several groups have increasingly implemented thalamic recording during SEEG (9). In parallel, the growing interest in thalamic DBS for refractory epilepsies highlighted the potential relevance of these recordings in stratifying DBS targets.

In the present work, we review the current use of thalamic SEEG and its interest in research and treatment of focal epilepsy.

## Methods

English-language and French-language articles related to thalamic SEEG were identified by a search in PubMed (1965–June 2024) using the following keywords: “thalamus,” “stereotactic techniques,” “intracranial EEG,” “electroencephalography” and “epilepsy.” The references in the selected papers were also included if relevant.

## Results

Based on the PubMed search, 67 articles were screened. Overall, after inclusion of the relevant references, the reported studies included 562 patients with SEEG recordings including thalamic implantation (1 to 121 patients/study).

### SEEG targets based on the anatomy of the thalamus

**Figure 1:**
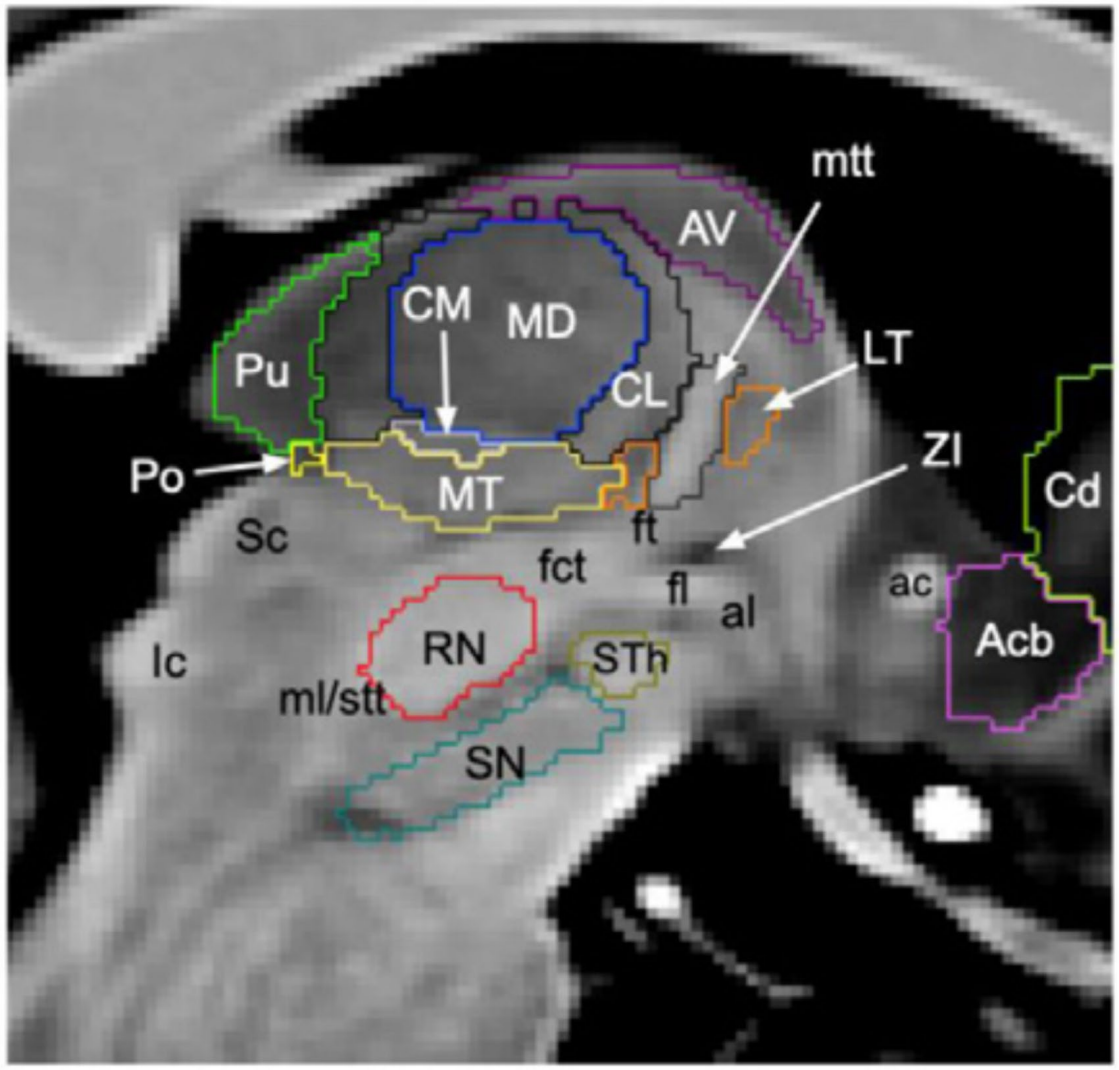
Segmentation of thalamic nuclei. Sagittal slice of T1-weighted 7 T brain magnetic resonance imaging where the thalamic nuclei most frequently implanted in SEEG are shown, i.e., pulvinar (Pu, green), centromedian (CM, grey), mediodorsal (MD, blue) and anterior nuclei (AV, purple). Figure from Brun et al. (78).

The thalamus consists of several nuclei (Figure 1) defined based on chemoarchitecture and cytoarchitecture (10) or connectivity (11) leading to multiple atlases with different numbers of defined nuclei (e.g., the stereotactic Morel atlas, cited here, involves 13 nuclei). However, thalamic nuclei have consistent topography and functionality across subjects (12). They can be subdivided into relay nuclei, the reticular nucleus and intralaminar nuclei (13). Among the relay nuclei, the anterior, mediodorsal, and pulvinar nuclei are associative nuclei and, thus, interesting targets for SEEG recordings due to their numerous connections to the limbic system, which is frequently involved in focal epilepsies (14). The reticular nucleus is a shell-shaped structure at the lateral part of the thalamus (15) that is difficult to explore by SEEG. Among the intralaminar nuclei, the centromedian nucleus is connected to widespread cortical areas. Regarding its implication in the seizure dynamics, it has been demonstrated that it can drive the cortex or, inversely, follow the cortex depending on the seizures (16). The aforementioned nuclei are the most frequent targets during SEEG recordings with thalamic implantation performed for fundamental and clinical research (4) using orthogonal transsylvian or posterior-to-anterior approaches (17). The pulvinar can be targeted without additional electrodes, by extending the trajectories of electrodes planned for cortical sampling in contrast with centromedian and anterior nuclei (6, 18, 19).

### Implication of the thalamus during seizure genesis, propagation and termination

**Figure 2:**
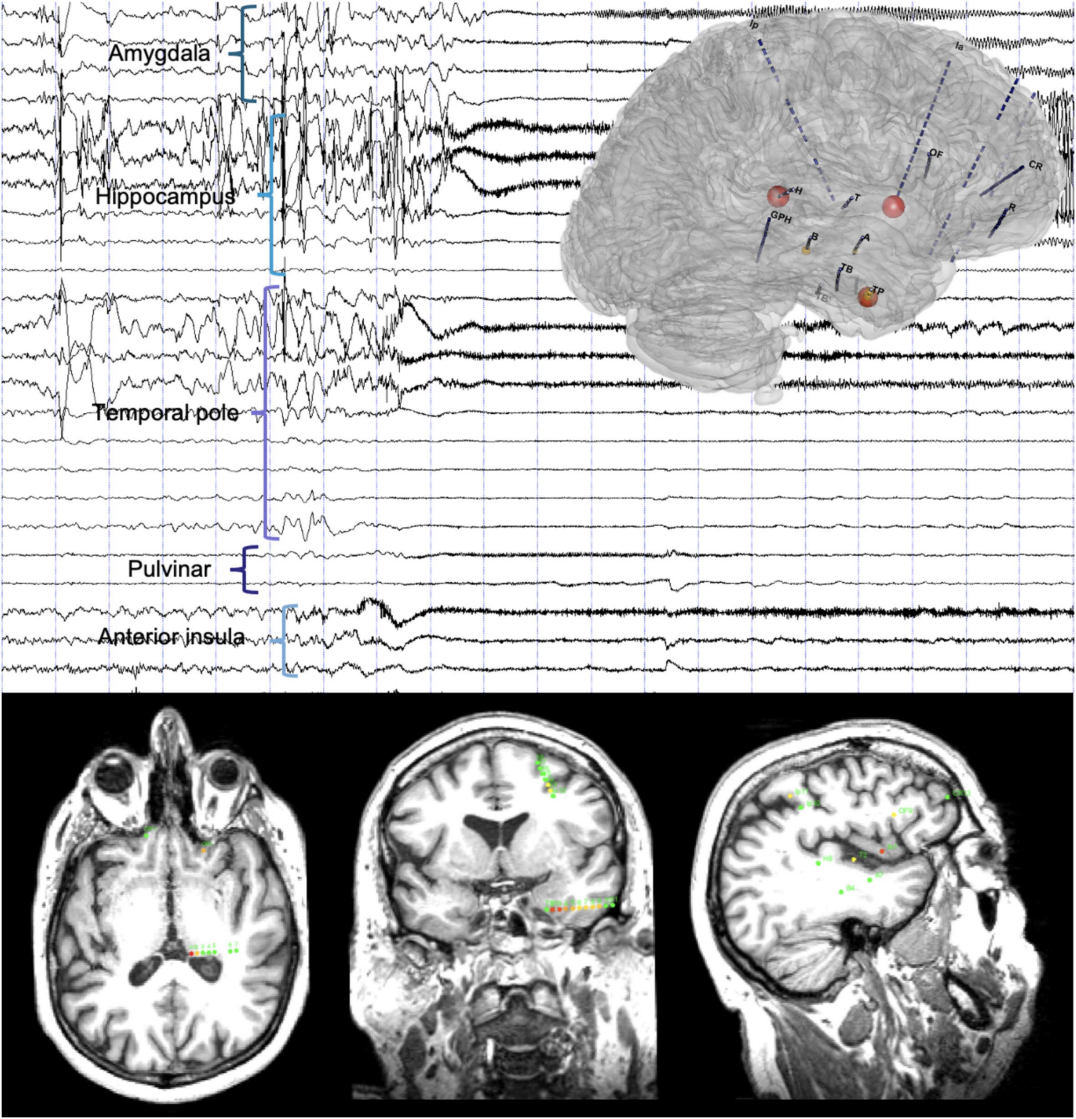
Timing of the thalamic implication in a temporal lobe seizure. Top: A 20-s SEEG recording dataset including a temporal lobe seizure and three-dimensional representation of the epileptogenicity index (EI). Seizure-onset is characterized by a DC-shift with low-voltage fast activity involving the anterior insula and the temporal pole, rapidly involving the pulvinar, and later amygdala and hippocampus. Bottom: Axial (left), coronal (middle) and sagittal (right) slices of T1-weighted brain MRI with a representation of the normalized EI values on the respective intracranial contacts (for details on the EI, see Bartolomei et al. (31), involved contacts represented according to a color scale from yellow to red, green contacts non involved in the discharge): Ia1-2 (anterior insula, right), EI = 1; H1-2 (pulvinar, left), EI = 0.97; TP2-3, TP3-4 and TP4-5 (temporal pole, middle), EI = 0.89 to 0.44.

Ictal SEEG recordings with available sampling of subcortical brain areas demonstrated thalamic implication during the first 15 s of the seizure in 86% of patients based on visual analysis (8, 20) (Figure 2).

**Figure 3:**
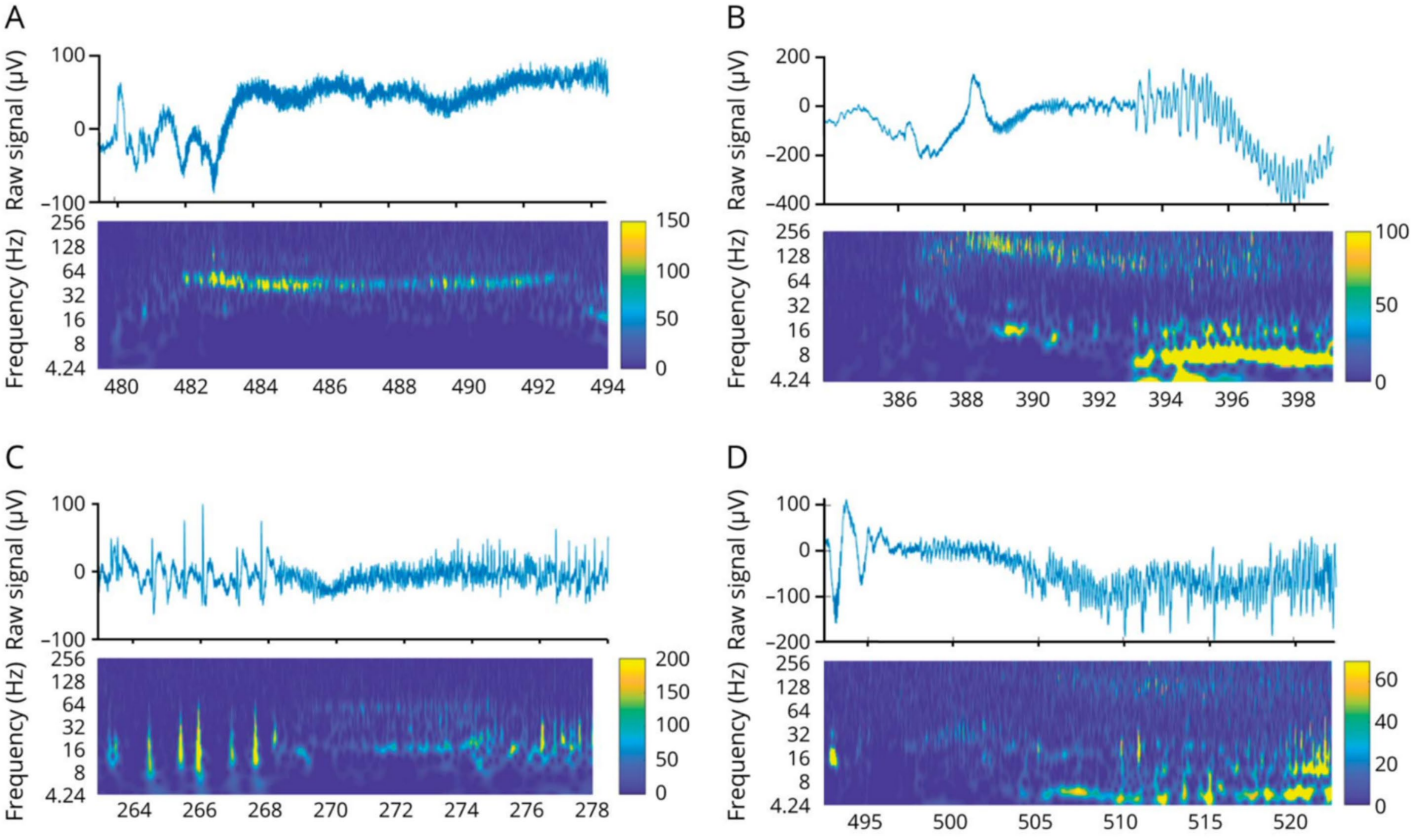
Thalamic seizure-onset patterns. **(A,B)** Low voltage fast activity. **(C)** Rhythmic spikes. **(D)** Theta discharge. Figure from Pizzo et al. (20).

Similarly to cortical regions (21), various patterns of seizure onsets were described at thalamic contacts, i.e., low-voltage fast activity (LVFA; 31%), rhythmic spikes (38%) or theta activity (18%) (8, 20) (Figure 3). High-frequency activity could be detected in all investigated thalamic nuclei, especially in the anterior and dorsomedial nuclei at the onset and in the centromedian nucleus at the end of the seizure (22). The sequential implication of the thalamic nuclei during the seizure time course is highly reproducible from one seizure to another, but cannot be predicted either based on the neuroanatomical knowledge, the cortical area where the seizure is starting, or the seizure semiology (23). The first thalamic ictal discharge can emerge before the first clinical manifestation (24).

The pulvinar implication in temporal lobe seizures is more frequent in medial temporal lobe epilepsy (mTLE) than in lateral temporal lobe epilepsy (lTLE) (7). Pulvinar LVFA occurs more frequently at the seizure onset in lTLE (73% vs. 27% in mTLE) and rhythmic repetitive spikes and slow waves occur more frequently in mTLE (77% vs. 23% in lTLE) (8). No specific thalamic pattern was detected during seizure propagation (8). However, thalamic SEEG could assess seizure propagation patterns within different sampled thalamic nuclei, and demonstrated a prominent implication of the pulvinar (25). Cortical LVFA at seizure onset is associated with a rapid propagation to the thalamus (22). Clinically, the early medial pulvinar implication in temporal seizures correlates with an early loss of consciousness during the seizure (7). The loss of consciousness was correlated with the level of thalamocortical synchronization, possibly due to the excessive synchronization within consciousness-related thalamic structures impeding information processing (26). Furthermore, it has been demonstrated that during wakefulness, the dorsomedial nucleus exhibits rhythmic gamma activity between 30 and 40 Hz that ceases during ictal propagation, simultaneously with the loss of consciousness, and reappears simultaneously with restoration of consciousness (27).

**Figure 4:**
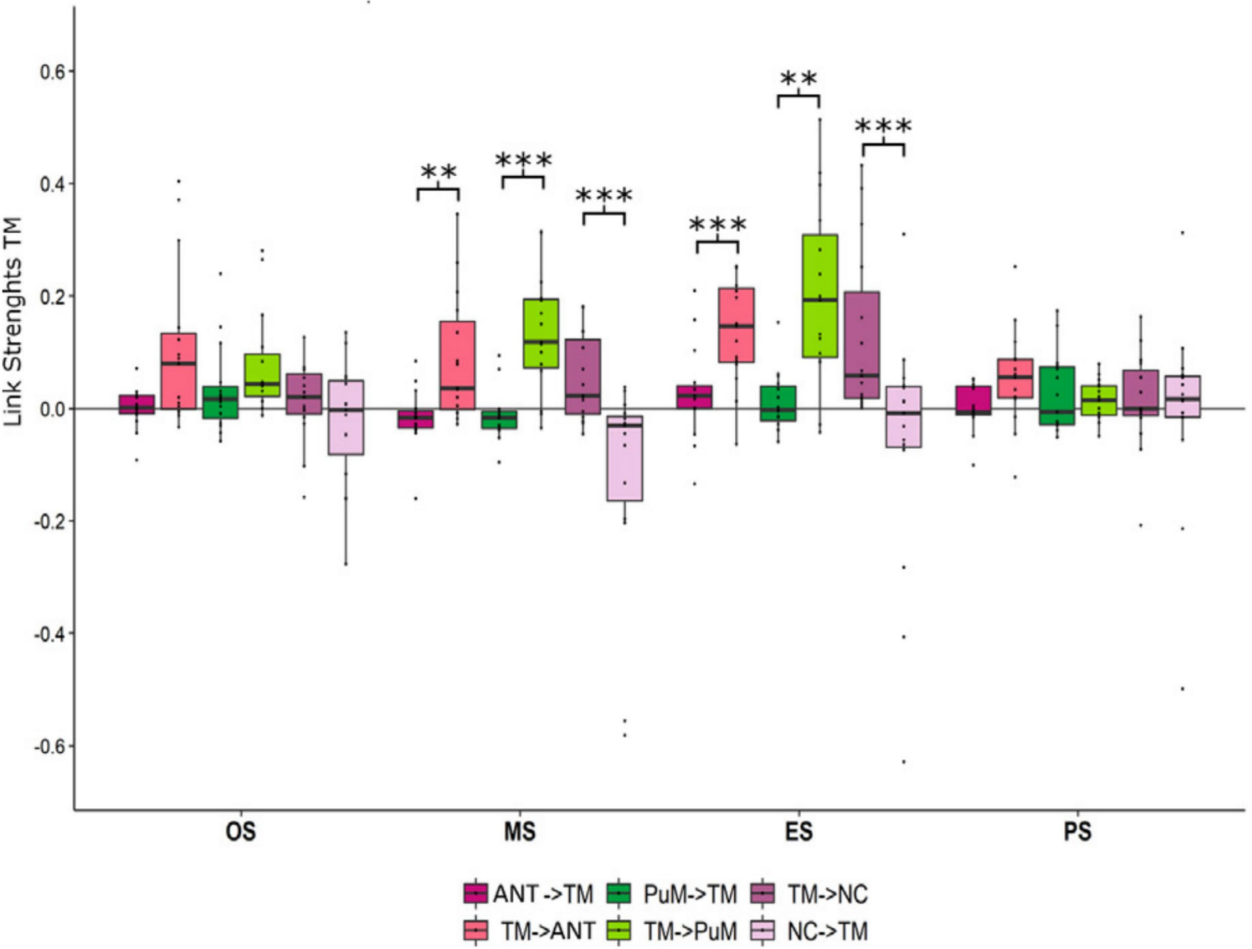
Afferences vs. efferences between the temporal regions and thalamic nuclei depending on the ictal timing. Mean and standard deviation of link strength between anterior nucleus (ANT), medial pulvinar (PuM), temporo-mesial structures (TM) and neocortical structures (NC) highlighting (i) stronger IN connectivity to the ANT at the middle (*p* < 0.01) and at the end (*p* < 0.001) of seizures than OUT connectivity, (ii) stronger IN connectivity to the PuM at the middle (*p* < 0.001) and at the end (*p* < 0.01) of seizures than OUT connectivity, (iii) stronger OUT connectivity from TM to NC at the middle (*p* < 0.001) and the end (*p* < 0.001) of seizures than IN connectivity. Figure from Soulier et al. (19).

Seizure termination, as observed through intracerebral EEG (iEEG), is characterized by two main patterns: synchronous, where neural activity ceases simultaneously across regions, and asynchronous, where cessation occurs in a more regionally staggered manner (28–30). Synchronous patterns are characterized by a higher thalamocortical synchronization at the end of seizure and more thalamic efferences than other seizures (29). Nevertheless, pulvinar and anterior nucleus are receivers of afferent information from the temporal lobe across the seizure (19) (Figure 4).

The estimation of ictal epileptogenicity of different brain structures using the epileptogenicity index (EI) (31) highlighted higher epileptogenicity values in the thalamic nuclei (implanted nuclei: pulvinar in 57% of patients, medial nucleus in 11%, lateral nucleus in 22%, not localized in 10%) compared to other subcortical structures, with the thalamus EI values as high as in the epileptogenic zone in 20% of cases (20). The thalamic epileptogenicity was correlated with the extent of the epileptogenic network (*ρ* = 0.32, *p* = 0.02) (20). Functional connectivity analysis highlighted increased thalamocortical connectivity at the seizure-onset of temporal seizures compared with the interictal period (7), followed by a subsequent increase at the end of the seizure (19). Connectivity did not significantly differ between two investigated thalamic nuclei (anterior nucleus and medial pulvinar), but the pulvinar seems to initiate the seizure cessation (19). Similarly to global cerebral synchronization, thalamic synchronization is higher at the end of a seizure compared to seizure onset (29). An increase in these global cerebral synchronization values was associated with a shorter seizure duration (*p* = 0.045), and thalamic synchronization followed the same tendency (*p* = 0.052) (29). Thalamic and cortical synchronization could lead to seizure cessation (32). The coherence between seizure onset and thalamic activity increases in the delta, theta, and alpha bands, and delta activity is mainly found at the centromedian nucleus during frontal lobe seizures and at the anterior nucleus during limbic seizures (33).

**Figure 5:**
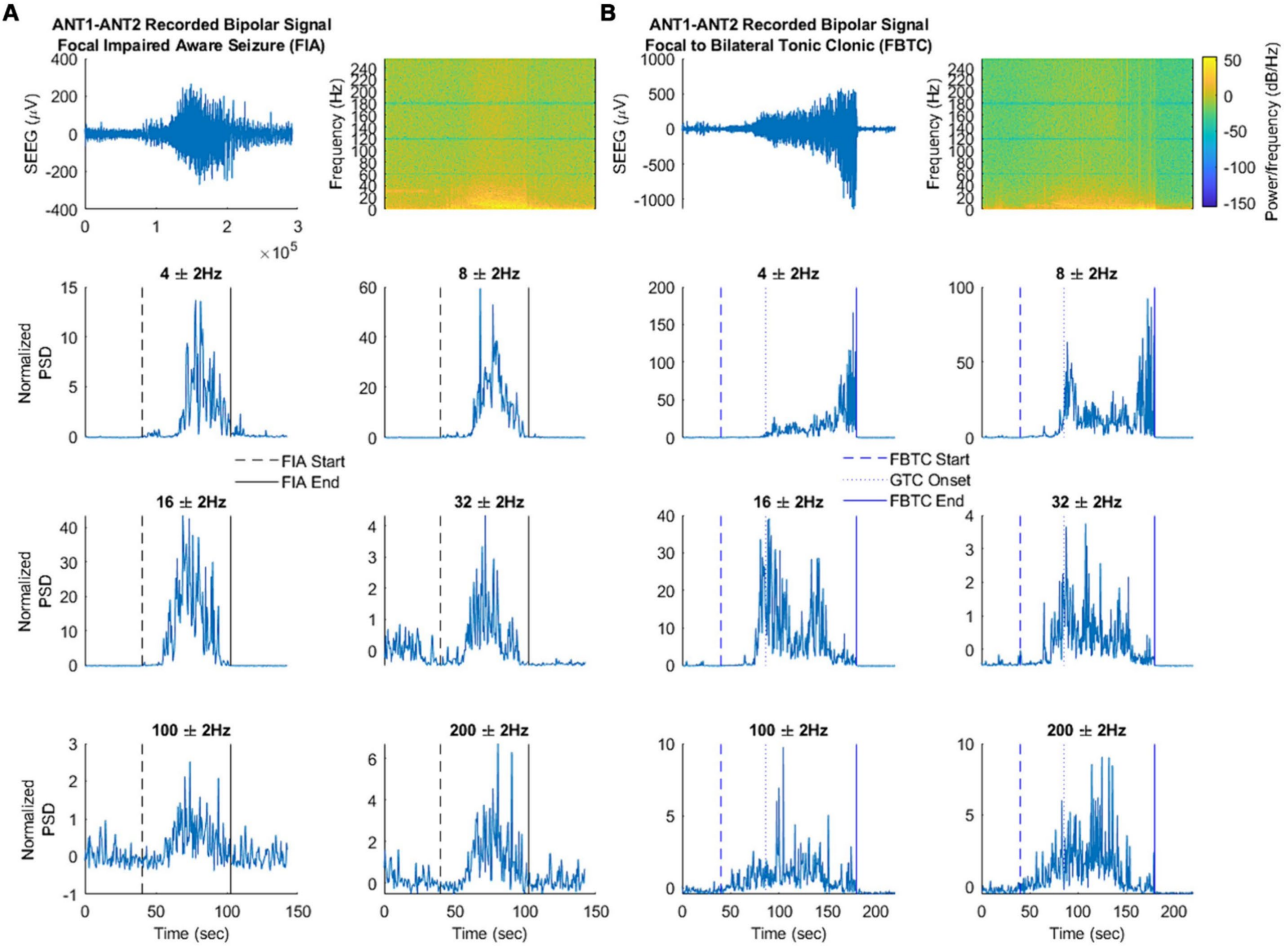
Frequency changes in the thalamic anterior nucleus during focal seizure with impaired awareness **(A)** vs. focal to bilateral tonic–clonic seizure **(B)**. Top: Filtered signal in the time domain and spectral analysis. Bottom: Signal power at each frequency of interest. Time-frequency analysis showed a large 2–6 Hz activity and a thin 8 Hz activity in the interictal period with a significant gain at 4 Hz during focal seizures with preserved consciousness and at 4 Hz, 8 Hz, 16 Hz during focal seizures with altered consciousness or secondary bilateralization. Moreover, an increase in 100–200 Hz activity occured during seizures with secondary bilateralization while a decrease at 32 Hz occurs during seizures with preserved consciousness. Figure from Singh et al. (34).

Electro-clinical seizures induced modification of local field potentials within the anterior nucleus, in contrast with electrical infraclinical seizures (34). Time-frequency analysis allows to discriminate focal seizures with preserved consciousness and those with altered consciousness or secondary bilateralization based on the frequency content of ictal activity (34) (Figure 5).

During the postictal period, it has been shown that the centromedian nucleus showed a postictal rhythmic 1.5–2.5 Hz delta activity simultaneously to the suppression of cortical background activity (35).

### Interictal thalamic recordings

The high rates of thalamic spikes/ripples and a high thalamocortical connectivity in the beta and gamma bands during sleep were related to poor surgical outcome (Engel class III/IV) (36).

### Thalamic stimulations

Electrical thalamic stimulation can induce various types of afterdischarges. However, electroclinical seizures triggered by thalamic stimulation are only exceptionally reported, e.g., mesiotemporal seizures induced by a 50 Hz stimulation of the midline thalamus (37). A 10 Hz stimulation increases cortical gamma activity, while a 50 Hz stimulation suppresses responses in the gamma band, however both those frequencies reduce the post-stimulation excitability (38) by either inhibition of upstream brain areas and short-term synaptic depression of thalamocortical connections in case of low-frequency stimulations, or inhibition of thalamic responses in case of high-frequency stimulations (39). Both hippocampi and neocortical brain areas with high epileptogenicity were more sensitive to this type of stimulation (38). Transient awareness alteration could be triggered by stimulations of the anterior thalamic nucleus or pulvinar, and was associated with a decreased functional connectivity (node strength) within the pulvinar as well as decrease in thalamo-cortical functional coupling (link strength), impacting connections with the insular, orbitofrontal, lateral prefrontal, temporal and parietal associative cortices (40). Interestingly, high-frequency stimulations of the centromedian nucleus during SEEG-recorded focal electro-clinical seizures were shown to discontinue seizures and associated ictal apnea (41), whereas 130 Hz stimulation of medial pulvinar could decrease seizure duration and loss of conciousness during SEEG-recorded temporal seizures, triggered by 50 Hz stimulation of the ipsilateral hippocampus (42).

Thalamo-cortical evoked potentials (TCEPs) following a pulvinar stimulation were observed in the operculo-insular areas in 90% of cases, in the lateral temporal areas in 78%, in the parietal cortex in 65%, in the frontal cortex in 52%, in the occipital cortex in 43%, in the mesiotemporal areas in 34% and in the cingular gyrus in 33% (43).

### Thalamic responses to cortical stimulations

Cortico-thalamic evoked potentials (CTEPs) were mainly observed within the pulvinar (23). CTEPs recorded in the pulvinar followed 80% of mesiotemporal stimulations, 76% of lateral temporal stimulations, 66% of cingular gyrus stimulations, 40% of parietal stimulations, 25% of occipital stimulations, 17% of frontal stimulations and 14% of operculo-insular stimulations (43).

Similarly to the results from CTEPS and TCEPs, a higher effective connectivity is described from the hippocampus to the thalamus than in the opposite direction (37). Additionally, the cortico-cortical evoked potentials (CCEPs) recorded within the posterior cingular gyrus following a hippocampal stimulation have a specific morphology related to their propagation in the Papez circuit through the anterior nucleus, that was confirmed by TCEPs of the anterior thalamus inducing an earlier but similar response (44).

The CCEPs analysis demonstrated a late evoked potential around 500 ms after stimulation in rodents via cortico-thalamo-cortical pathways (45). It was therefore assumed that the described third peak in human CCEPs also evidences cortico-thalamo-cortical connectivity (46). Furthermore, the increased latency (46) and the increased variability (47) of this third CCEP peak within the epileptogenic zone could be the interictal correlate of thalamic involvement in the epileptogenic network. The transmission via electrical synapses could induce the third peak of the CCEPs (46). Moreover, a higher proportion of subcortical responses are elicited after a single-pulse electrical stimulation within the epileptogenic zone (48).

### Prediction of the surgical and neuromodulation outcome

Estimating ictal and interictal epileptogenicity biomarkers is important for the prognostication of surgical outcome (49, 50) and might help to limit epilepsy surgery failures (51). High ictal thalamic epileptogenicity values, as estimated by the EI (EI > 0.3), were associated with a poorer surgical outcome at 1 year (20). Thus, thalamic EI values assessed from ictal SEEG recordings could be a potential biomarker of the postoperative prognosis (20). Similarly, increased thalamocortical coupling at the seizure onset was related to a poor surgical outcome (7). Moreover, thalamocortical connectivity was demonstrated to be predictive of the neuromodulation response in focal epilepsy in several recent studies (36, 52, 53). Finally, greater functional connectivity between the seizure sites and the DBS site correlated with more favorable outcome of DBS (54).

A better understanding of the seizure cessation mechanisms could help to select more appropriate DBS targets and/or stimulation parameters (19, 55, 56). Indeed, one third of patients are not responsive to the anterior nucleus DBS. It has been suggested that this failure could be due to the early implication of other thalamic nuclei in the ictal discharge (23). The pulvinar represents a promising target for DBS due to its role in the cessation of seizures (19). As already mentioned above, medial pulvinar stimulations applied during the SEEG recorded temporal seizures could reduce seizure duration and ictal consciousness alteration (42).

Cortico-cortical connectivity modifications induced by thalamic stimulation could be used as a biomarker for the DBS efficacy (36, 53, 57–59). A prolonged thalamic stimulation (≥90 min) at a high frequency (145 Hz) reduced the amplitude of distant CCEPs, showing a modulation of the distant connectivity (58). Disruption of the ictal discharges (i.e., mainly low-voltage fast activity commonly occurring within the first 400 ms in 95% of seizures) occur after thalamic responsive neurostimulation (RNS) (60). Nonetheless, the mechanism that underlies the efficacy depending on the stimulation target is not well understood (61). The development of algorithms to detect seizures during thalamic SEEG would allow to develop closed-loop thalamic stimulation (62).

### Deep brain and responsive stimulation recordings of thalamic activity

DBS and RNS recordings also help to better understand the timing of thalamic involvement. Such recordings linked the evolution to bilateral tonic–clonic seizures with the centromedian nucleus involvement in patients with focal epilepsy (63). It was shown that during generalized seizures thalamic activity precedes cortical activity whereas thalamic activity follows cortical discharge in frontal seizures (16). The same study demonstrated presence of independent focal unilateral epileptiform discharges restricted to the centromedian nucleus suggesting the possibility of autonomous ictogenesis by these thalamic structures (16). Other studies compared the results of thalamic and scalp EEG recordings and demonstrated that discharges in the anterior nucleus were observed in patients disclosing bilateral scalp EEG discharges, whereas discharges in the dorsomedial nucleus were seen in patients presenting with unilateral scalp EEG discharges (64).

A few recent studies attempted to demonstrate potential benefit of better stratifying the stimulation target depending on electro-clinical features. In particular, centromedian nucleus DBS reduced the generalized seizure frequency (65) while having a little effect on focal seizures (66), anterior nucleus DBS reduced temporal lobe seizures (59), whereas pulvinar DBS has been shown to reduces seizure frequency in different subtypes of focal drug resistant epilepsy not accessible to surgery or after failure of resective surgery (67, 68). Another recent study has suggested that thalamic nucleus to be stimulated might be chosen depending on the type of epilepsy (69). Furthermore, multisite thalamic stimulation could be considered (70).

### Risk–benefit ratio and safety considerations

Few studies reported rare short-term complications following thalamic implantation (4), especially symptomatic hemorrhages not in the thalamic area (71). Robot-assisted procedures improve the safety and precision of implantation allowing to reach the thalamic targets using conventional SEEG trajectories without adding supplementary electrodes (72). Yet the risk–benefit ratio may vary across the targeted nuclei and should be particularly carefully evaluated when targeting centromedian, dorsomedial and anterior nuclei (6). To date, the hemorrhagic complications reported in patients with thalamic coverage have not occurred within the thalamic areas and could be regarded as possibly unrelated to the thalamic targeting, *per se.*

## Discussion and conclusion

SEEG implantation eventually including thalamic targets always involves surgical risks, therefore risk–benefit ratio should be carefully considered (72). Still, no percentage of additional risk related to thalamic implantation is provided in published studies. Nonetheless, there is a large body of evidence for a safety of conventional thalamic SEEG targets. Thalamic recordings provide valuable insides into pathophysiology of focal epilepsies, in particular the ictogenesis and epileptogenesis, and help to better estimate the global organization of the epileptogenic networks in a given patient (6). The existing literature data underline the importance of studying both cortical and subcortical brain areas that may be synchronously involved in epileptogenic networks (7). The early and/or prominent involvement of the thalamus is associated with worser epilepsy surgery outcome (7), specifically in cases of bitemporal epilepsy. Thus, estimating the thalamic epileptogenicity during the SEEG recording may be valuable for predicting surgical outcome (20). While this can be estimated with only one implanted thalamic nucleus (20), the evaluation of neurostimulation targets during SEEG should require the implantation of multiple nuclei. Through stimulation and recording of different thalamic nuclei, SEEG can provide helpful information to guide target selection for personalized neuromodulation strategies using model-free (72, 73) and model-based approaches (74). Targeting the medial pulvinar is relatively straightforward. It can be accessed by inserting the superior temporal gyrus electrode deeper (6). In our view, the medial pulvinar should be almost systematically targeted when a single orthogonal trajectory is used to sample the planum temporale, Heschl’s gyrus, and the posterior inferior insular cortex. These areas would require electrode implantation regardless, and reaching the posterior thalamus would require only a modest extension of the planned trajectory. In contrast, targeting the centromedian or anterior thalamic nucleus often necessitates a dedicated oblique trajectory. These nuclei—especially the anterior thalamus—are not easily accessible through a standard orthogonal approach. Their inclusion should therefore be considered on a case-by-case basis, informed by preoperative noninvasive data. This is all the more significant given the absence of definitive evidence that thalamic involvement observed on SEEG reliably predicts the therapeutic response to DBS. The cortical entry point should lie within the putative epileptogenic zone, and the trajectory should ideally extend from already sampled regions. Such approaches should remain within academic epilepsy surgery centers, preferably within clinical research protocols, discussed in multidisciplinary team meetings, and supported by the patient’s comprehensive informed consent. The recent technique from the Stanford group (17) offers an elegant solution by enabling the recording of multiple thalamic nuclei with a single lead. This strategy facilitates personalized mapping of seizure propagation networks through the thalamus. It should be reserved for carefully selected cases in experienced centers, particularly when preoperative investigations suggest resective surgery is unlikely and when thalamic recordings are expected to yield relevant data for neuromodulation planning. Finally, additional thalamic sampling requires specific MRI protocols to visualize the relevant nuclei accurately. Routine MRI sequences used in standard SEEG planning may be insufficient. Sequences such as STIR, f-GATIR, or QSM have demonstrated improved delineation of thalamic nuclei (75–77).

To conclude, the thalamic implantation scheme, including the number of implanted thalamic nuclei, should be defined based on the questions that are to be answered during SEEG.
